# Development of biomarker panel to predict, prevent and create treatments tailored to the persons with human papillomavirus-induced cervical precancerous lesions

**DOI:** 10.1186/1878-5085-5-1

**Published:** 2014-01-06

**Authors:** Liudmyla M Lazarenko, Olena E Nikitina, Evgen V Nikitin, Olga M Demchenko, Galyna V Kovtonyuk, Larysa O Ganova, Rostyslav V Bubnov, Veronika O Shevchuk, Natalia M Nastradina, Viktoria V Bila, Mykola Ya Spivak

**Affiliations:** 1 Zabolotny Institute of Microbiology and Virology, National Academy of Sciences of Ukraine, Zabolotny str. 154, Kyiv 03680, Ukraine; 2 Odessa National Medical University, Ministry of Health of Ukraine, Odessa 270039, Ukraine; 3 JSC SPC ‘DiaprofMed’, Svitlycky str. 35, Kyiv 04123, Ukraine; 4 Perinatal Center, Kyiv, Ukraine, Kotelnikova str. 95, Kyiv 03179, Ukraine; 5 Clinical Hospital ‘Pheophania’ of State Affairs Department, Zabolotny str., 21, Kyiv 03680, Ukraine

**Keywords:** Predictive, Preventive, Personalized medicine, Pro- and anti-inflammatory cytokines, Papillomavirus, Herpes simplex virus infections, Cervical precancerous lesions, HPV-induced cervical precancerous diseases, Pro- and anti-inflammatory cytokines, Antibodies, Avidity, Immunomodulators, Ultrasonography, Biomarker panel

## Abstract

**Introduction:**

Human papillomavirus (HPV) induce many cancer conditions and cause cervical cancer, second in frequency of malignant disease in women.

The aim was to develop biomarker panel for HPV-induced cervical precancerous diseases in patients infected with herpes simplex virus (HSV).

**Material and methods:**

The study involved 71 women with cervical precancerous diseases (mean age 26 ± 5 years) revealed by colposcopic, cytomorphological, and ultrasound signs which were assessed according to the following: first group, 44 patients infected with HPV; second group, 27 HPV-negative patients; and third group, 30 healthy patients (controls). In cervical specimen, we identified HPV DNA of different oncogenic risk types by polymerase chain reaction (PCR). Enzyme-linked immunosorbent assay (ELISA) kits (JSC SPC ‘DiaprofMed’) were used for detecting antibodies to HSV1 and/or HSV2 and for determining the avidity index. The production of pro-inflammatory cytokines, interferon-γ (IFN-γ), IFN-α, TNF-α, and interleukin-1β (IL-1β), and anti-inflammatory cytokines, IL-4, IL-10, and transforming growth factor-β1 (TGF-β1), were studied by ELISA.

**Results:**

In HPV-induced cervix precancerous diseases, we identified low-avidity IgG antibodies to HSV serum of 20 patients; in the serum of 17 patients, we identified average-avidity antibodies, and high-avidity antibodies were found in 2 patients only. In 14 HPV-negative patients, we found low-avidity IgG antibodies to HSV; in 10 patients, medium avidity. Patients with low-avidity IgG antibodies to herpes virus showed high and medium oncogenic risk HPV types and a decrease of IFN-γ compared to patients with medium-avidity IgG antibodies. Production of IFN-γ was suppressed also in HPV-negative patients with cervical precancers, but we found low- and medium-avidity IgG antibodies to herpes virus. In patients with low-avidity antibodies, we observed increased level of IL-10. Level of IFN-α, IL-1β, IL-2, and IL-4 did not change in patients of all groups, but TGF-β1 increased.

**Conclusions:**

In HPV-positive patients, those with low-avidity IgG antibodies to HSV had immunosuppression, confirmed by increased TGF-β1 and violation of IFN-γ production. Therefore, in pro- and anti-inflammatory cytokines and IgG antibodies to HSV, their avidity is an important diagnostic biomarker of HPV-induced precancerous cervical diseases. Low-avidity IgG antibodies may be an indication for treatment with immunomodulators and antiviral drugs.

## Overview

### Predictive, preventive and personalized medicine in cervical cancer

Women health and gender-related pathology is among the priorities for predictive, preventive, personalized medicine (PPPM) [1-4] - an innovative approach towards gynecology cancer prevention that aims at detection of pre-malignant stages for novel integrative cancer management including development predictive schemes followed by tailored prevention with implementation of personalized treatment strategies [3]. *Cancer of the cervix* (cervical cancer, CC) is the second most common cancer in women worldwide, with about 500,000 new cases and 250,000 deaths each year [5].

*Human papillomavirus* (HPV), which induces a wide range of diseases and precancerous tumor genesis, is the most important risk factor for cervical cancer. The interaction of oncoproteins E6 and E7 of high oncogenic risk with intracellular factors is a key stage for induction of malignant transformation. These factors play an important role in the regulation of growth, differentiation and apoptosis, which may lead to instability of the genome and malignant transformation [6-9].

The risk co-factors of HPV-induced cancer are as follows:

• Patients infected by other sexually transmitted pathogens, especially viruses like herpes simplex virus (HSV) [2];

• The violation of a specific cellular immune response [8-11] and production of Th1-type cytokines, etc. [12-17];

• HPV type, especially in the case of cervical infection;

• Frequent multiple full-term pregnancies and birth giving before 16 years;

• Congenital and/or acquired immunosuppression;

• Patients infected with HSV-2;

• Use of steroids (dexamethasone, progesterone and estrogen and corticosteroids), oral contraceptives;

• Genetic factors - polymorphism of the E2 protein gene of HPV and molecules of the major histocompatibility complex (MHC), interferon-γ (IFN-γ), tumor necrosis factor-α (TNF-α) and interleukin-1β (IL-1β);

• Smoking, lack of antioxidants in the body or folic acid due to low socio-economic standard of living, etc.

It was noted that E6 and E7 oncoproteins of high oncogenic risk HPV have immunosuppressive activity directed primarily to the inhibition of gene expression of IFN and IFN-induced genes, as well as decrease in the production of IL-18, which directly regulates γ-interferonogenesis. This in turn affects the balance of cytokines Th1 and Th2 type and thus determines the main path of development of the immune response - either by Th1 or by Th2 type [9,18,19]. The carcinogenesis in HPV infection also involves Th3-type cytokine, including anti-inflammatory transforming growth factor-β (TGF-β) [20,21].

The role of pro-inflammatory cytokines is ambiguous, which can affect both the formation of the body’s immune defence in the HPV [9,22-24] and enhance the growth of transformed cells, as shown *in vitro*[18]. We have previously found that in cervical cancer patients with HPV with severe cervical intraepithelial neoplasia (CIN), the production of IFN-γ and IFN-α was suppressed and the production of pro-inflammatory cytokines and TGF-β increased [15]. Possibly, an imbalance of pro- and anti-inflammatory production of cytokine may be a risk factor for HPV-induced malignancies and underlie cervical cancer relapse in HPVI.

The issue of the role of HSV-1 and HSV-2 as co-factors of HPV-induced carcinogenesis was debated for a long time since 1982 [6]. HSV can increase the replication of high oncogenic risk HPV and its integration into the genome of the host cell [19], enhancing the expression of oncogenes E6 and E7, that is considered a relevant prerequisite for HPV-induced malignant transformation [25,26].

The results of epidemiological studies [27,28] also support the importance of role of HSV as a possible co-factor of HPV-induced cervical cancer. However, it was concluded that infection with HSV is not an obligatory factor for maintaining the transformed phenotype of cells in HPV-induced cancers [29,30]. In favor of co-factor mechanism of HSV-2 supported with the fact that the HPV-induced cervical adenocarcinoma, HPV DNA was not integrated into the genome of transformed cells transport in any case [29].

We have previously reported [31] that in the serum of most patients with HPV-induced cervical dysplasia, class G antibodies to HSV-1 and/or HSV-2 were found, which had mostly low or medium avidity index, which indicates on the final stage of primary infection, and the presence of chronic recurrent infection. In patients with low-avidity antibodies to HSV-1 and/or HSV-2, CIN was diagnosed in major cases than in patients with antibodies with medium avidity to these viruses. However, it remains still unknown how HSV can function as a co-factor for HPV-induced CC.

The aim was to assess the production of pro- and anti-inflammatory cytokines in HPV-induced cervical precancerous diseases in patients infected with HSV-1 and/or HSV-2 in serum IgG, identified with varying degrees of avidity, specific to these HSV.

## Methods

### Patient’s inclusion and sample collection

The study involved 71 women with cervical precancerous diseases (mean age 26 ± 5 years). Human papillomavirus infection and cervical precancerous diseases were diagnosed on the basis of colposcopic [32] and cytomorphological [33,34] and ultrasound (US) data. The survey was conducted at the Department of Obstetrics and Gynecology, Odessa National University (Ukraine).

All patients underwent general clinical examination, which included clinical and biochemical blood tests, blood tests for HIV, RW, HBS-Ag and HCV-Ag, clinical urine tests, ECG, ultrasound, chest X-rays and a study of vaginal biotope (microflora), according to the protocols of the Ministry of Health of Ukraine.

**Figure 1:**
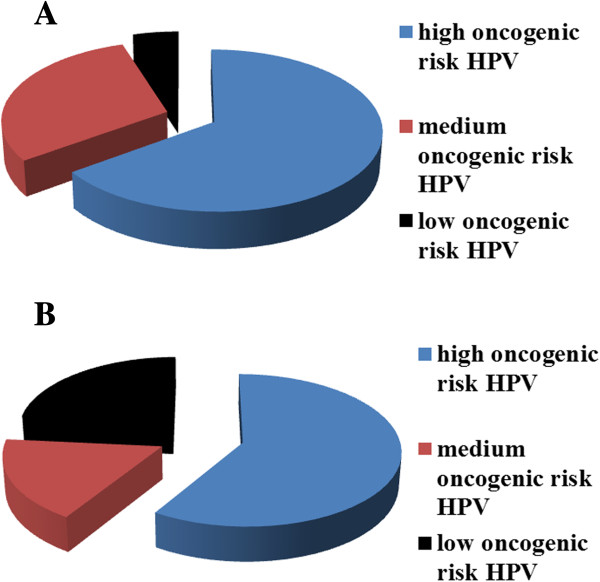
**Cytology of cervical epithelium, HPV-induced CIN I.** Binuclear koilocytes with enlightenment zone around the nucleus, which arises from the cytopathic action of the virus, with a dense basophilic cytoplasm in a state of vacuole dystrophy. Painted by Pappenheim (×100 magnification).

**Figure 2:**
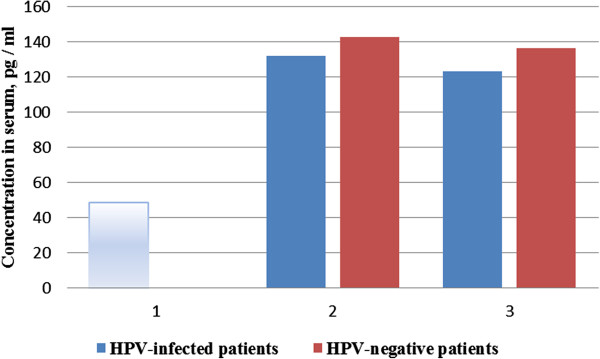
**Cytology of cervical epithelium, HPV-induced CIN II.** Binuclear koilocytes with signs of atypia. Painted by Pappenheim (×100 magnification).

**Figure 3:**
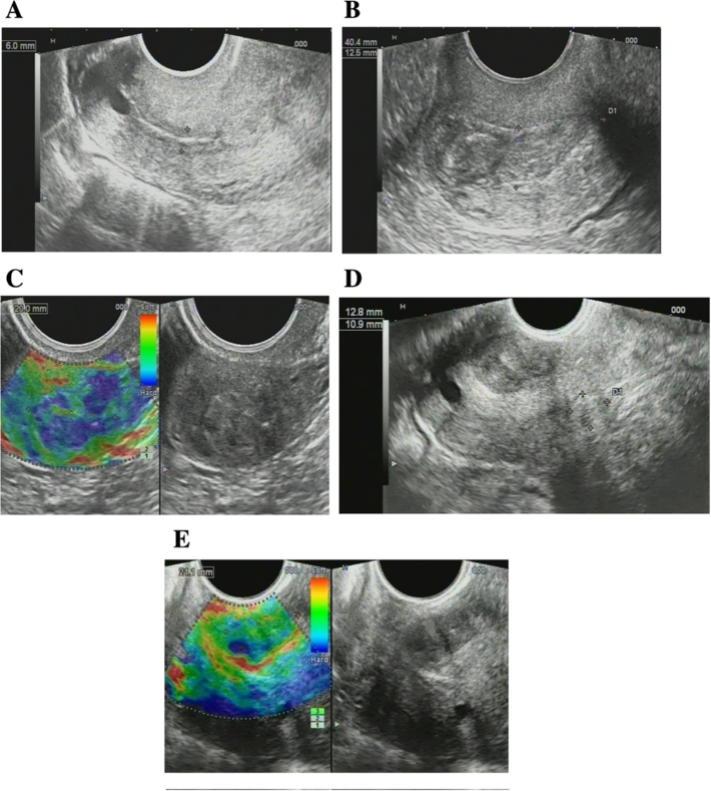
**Cytology of cervical epithelium, HPV induced CIN II.** Squamous cells with enlarged nucleus. Painted by Pappenheim (×100 magnification).

**Figure 4:**
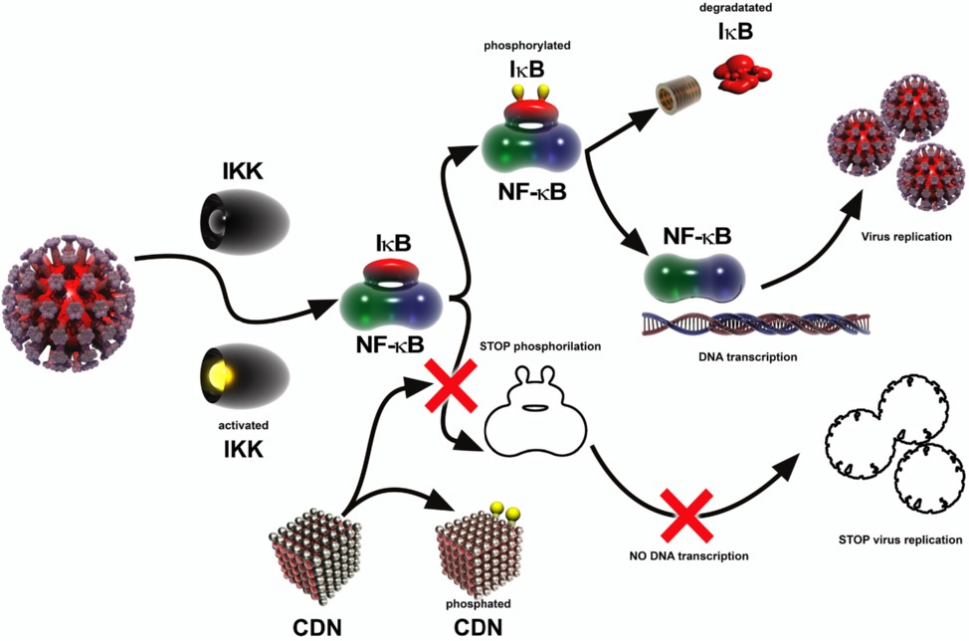
**Cytological examination of material cervix, HPV-induced CIN III. (A)** Binuclear koilocytes. **(B)** Multinucleated cells with signs of atypia. Painted by Pappenheim (×100 magnification).

According to the nature of the pathological process in cervix, we formed the following groups of patients with cervix precancerous diseases: (1) 44 patients identified HPV DNA in cervical specimen with colposcopic, cytomorphological (Figures 1, 2, 3 and 4), molecular and ultrasound signs of cervical precancerous diseases; (2) 27 patients, whom HPV DNA in cervical specimen were not identified but showing colposcopic, cytomorphological and ultrasound signs of cervical precancerous diseases and (3) the control group included 30 healthy women.

The patients were distributed into the groups with non-significant difference among groups as regards to age.

The design of the study was *prospective, non-randomized.*

From the patients and clinically healthy women, non-heparinized peripheral blood (20 ml) was taken to obtain the serum.

### HPV typing

DNA HPV detected by polymerase chain reaction (PCR) in cervical specimen. The HPV DNA of different oncogenic types: high (HPV 16, 18, 45, 56), medium (HPV 31, 33, 35, 51, 52, 58) and low (HPV 6, 11, 42, 43, 44).

### ELISA for detecting antibodies to HSV-1 and/or HSV-2 and determination of avidity index

For screening the sera for IgG for presence of HSV, we used the test system ‘DIA-HSV-1/-2-IgG’, constructed in the form of indirect solid phase enzyme immunoassay (ELISA). The solid phase polystyrene plates PolySorp (Nunc, Denmark) were used, which adsorbed the mixture of recombinant proteins gG1 and 2 gG (JSC SPC ‘DiaprofMed’, Ukraine). Murine monoclonal antibodies to human IgG labelled with horseradish peroxidase were used as a conjugate. TMB reaction, diluted in citrate buffer containing hydrogen peroxide, was used as a developer.

Differential diagnosis for HSV-1 and HSV-2 was performed using kits manufactured in the same format as above. However, in the immunosorbent, only the recombinant proteins gG1 or gG2 were used respectively. Based on the last test, the system was designed such that it enables not only to detect IgG to HSV but also to determine the degree of avidity.

The avidity index was calculated as the percentage of absorbance obtained in the test sample in the presence of a dissociating agent; the absorbance was obtained in its analysis as usual regimen. Thus, if avidity index was less than 30%, we supposed that serum contains low-avidity antibodies, in the range of 30% to 60%, it contains medium-avidity antibodies, and if more than 60%, high avidity. The setting reaction was conducted according to the manufacturer’s instructions for these kits (JSC SPC ‘DiaprofMed’).

### Cytokine analysis

The production of pro-inflammatory cytokines, IFN-γ, IFN-α, TNF-α, IL-1β and anti-inflammatory cytokines, IL-4, IL-10, TGF-β1, in determining the levels of these cytokines in serum of patients was studied by ELISA. To determine the serum TGF-β1, we used test production system User’s Manual (DRG Instruments GmbH, Germany). The levels of IFN-γ, IFN-α, TNF-α, IL-1β, IL-4 and IL-10 in serum were determined using appropriate ELISA test kits of Vector-Best (Russian Federation). Setting ELISA was performed according to the manufacturer’s instructions specified test systems.

### Statistical analysis

These data were processed by a computer program STATISTICA. The null hypothesis for the control and experimental groups tested using non-parametric Kolmorogov-Smirnov test. Data was presented as M ± SEM. Some experimental results are presented as median and interquartile range MAE (LQ − UQ), where Me is the median, LQ and UQ are the lower and upper quartiles, respectively. The significance level for all tests was 5% (*p* = 0.05).

The written informed consent for research was obtained from all patients. The medical ethics commissions of the Odessa National Medical University approved the study.

### Study limitation

The study was non-randomized, non-blinded. We are aware of small numbers made; it was difficult to exclude selection bias and information bias: the patients were motivated to participate in these studies because they had the access to their diagnostic profile that determined a tactic of personalized treatment using immunomodulators and antiviral drugs. Due to technological and financial limitations, we were not able to evaluate the extensive panel of the existing biomarkers to suggest reliable predictive program. Serum and imaging (US) biomarkers were assessed on small group of patients. For this reason, biomarker specificity/sensitivity was not evaluated as well as the measurements of individual outcomes were not sufficiently assessed according to the findings of the study.

## Results

### Groups of patients and characterization of the pathological process

In HPV-induced cervical precancerous diseases, we identified IgG antibodies to HSV-1 and/or HSV-2 with low avidity in serum of 20 patients; in serum of 17 patients, we identified IgG antibodies to HSV-1 and/or HSV-2 with average avidity, and high-avidity IgG antibodies to these HVS were found in serum of 2 patients only. Antibodies to HSV-1 and/or HSV-2 were not found in serum of two HPV-positive patients.

In the serum of 14 HPV-negative patients, we revealed IgG antibodies to HSV-1 and/or HSV-2 with low avidity, and serum of 11 patients in this group had IgG antibodies to HSV-1 and/or HSV-2 with a medium avidity; high-avidity IgG antibodies to these HVS were found in serum of 1 patient only. Antibodies to HSV-1 and/or HSV-2 were not found in the serum of two HPV-negative patients.

**Figure 5:**
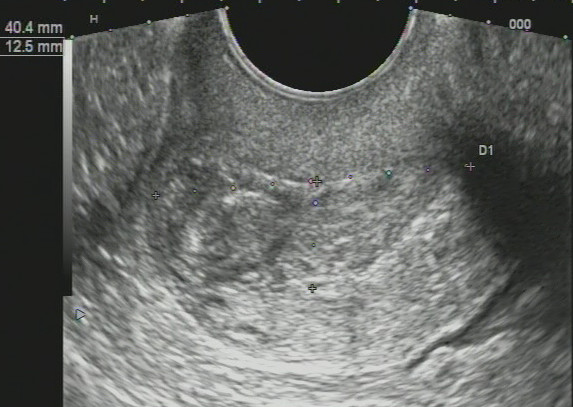
**Frequency of HPV infection of high, medium, and low oncogenic risk of patients with cervical precancerous diseases.** Precancer conditions of low- **(A)** and medium-avidity **(B)** IgG antibodies to HSV-1 and/or HSV-2 in serum (data presented in %).

In HPV-induced cervical precancerous diseases, we detected HPV of high oncogenic risk (HPV 16, 18, 45, 56), medium (HPV 31, 33, 35, 51, 52, 58) and low (HPV 6, 11, 42, 43, 44) (Figure 5). Thus, in 13 of 20 patients (65.0% cases) with low-avidity IgG antibodies to HSV-1 and/or HSV-2, we identified high-risk HPV types (2 patients had also medium risk HPV types). In six patients (30.0% cases) with low-avidity IgG antibodies to HSV-1 and/or HSV-2, medium-risk HPV types were identified. In one patient only (5.0% cases) with low-avidity IgG antibodies to HSV-1 and/or HSV-2, low-risk HPV types were identified.

In 10 of 17 patients (58.9% cases) with medium-avidity antibodies to HSV-1 and/or HSV-2, high-risk HPV types were identified (4 patients were also medium-risk HPV types). In 3 patients (17.6% cases) with medium-avidity antibodies to HSV-1 and/or HSV-2, medium-risk HPV types were detected. Low-risk HPV types was identified in four HPV-positive patients (23.5% cases) with low avidity antibodies to HSV-1 and/or HSV-2.

Thus, high- and medium-risk HPV types were more common in patients with low-avidity IgG antibodies to HSV-1 and/or HSV-2 (95.0% of cases) than in patients with medium-avidity IgG antibodies to HSV (76.5% of cases) in serum.

Two patients with high-avidity antibodies to HSV were infected with high-risk HPV types.

### Production of pro-inflammatory cytokines

The study of production of pro-inflammatory cytokines IFN-α, IFN-γ, IL-1β, IL-2, and TNF-α in patients with cervical precancerous diseases with low or medium-avidity serum IgG antibodies to HSV-1 and/or HSV-2 is presented in Table 1.

**Table 1:** Serum cytokine levels in patients with cervical precancerous diseases with low or medium-avidity IgG antibodies to HSV-1 and/or HSV-2

**Groups of people surveyed**	**Avidity antibodies to HSV**	**The concentration of cytokines pg/ml (M ± SEM)**				
		**IFN-α**	**IFN-γ**	**IL-2**	**TNF-α**	**IL-1β**
Clinically healthy	-	71.30 ± 32.51	32.50 ± 13.25	7.08 ± 0.98	8.10 ± 1.60	41.80 ± 4.51
HPV-positive patients	Low avid	96.27 ± 61.91	11.12 ± 5.31***	10.44 ± 6.21	10.60 ± 6.8	43.93 ± 20.86
	Middle avid	109.07 ± 78.52	25.47 ± 19.6	6.87 ± 1.60	8.68 ± 5.40	44.64 ± 18.43
HPV-negative patients	Low avid	79.10 ± 25.59	9.42 ± 4.47*	8.39 ± 1.79	9.56 ± 5.38	43.00 ± 17.32
	Middle avid	63.14 ± 25.53	11.69 ± 8.12**	8.46 ± 1.94	5.38 ± 1.34	42.98 ± 5.45

*p < 0.05 compared with control; **p < 0.025 compared with control; ***p < 0.025 compared with patients with medium-avidity antibodies to HSV-1 and/or HSV-2.

We found that IFN-α and IL-1β, IL-2 and TNF-α production at the system level was not changed in HPV-positive and HPV-negative patients with cervical precancerous diseases, who had low or medium-avidity IgG antibodies in serum to HSV-1 and/or HSV-2. Thus, in these patients, the serum levels of these cytokines were similar to that of the control group (clinically healthy women).

However, serum level of IFN-γ significantly decreased in HPV-positive patients with low-avidity IgG antibodies to HSV-1 and/or HSV-2 comparing to controls as well as HPV-positive patients with medium-avidity IgG antibodies to HSV-1 and/or HSV-2. It should be noted that serum level of IFN-γ decreased slightly in HPV-positive patients with medium-avidity IgG antibodies to HSV-1 and/or HSV-2, but the difference compared with the controls was not statistical.

Serum IFN-γ was also significantly lower in HPV-negative patients with cervical precancerous diseases with both low and medium-avidity IgG antibodies to HSV-1 and/or HSV-2 than in controls.

These data suggest that production of IFN-γ was suppressed in HPV-positive patients with low-avidity IgG antibodies to HSV-1 and/or HSV-2 as well as in HPV-negative patients with cervical precancerous conditions of low or medium-avidity antibodies to these herpes virus. However, there are no dependencies between the level of serum IFN-α, IL-1β, IL-2 and IL-4 in the two groups of patients in comparing the presence of serum with low- and medium-avidity or IgG antibodies to HSV-1 and/or HSV-2.

### Production of anti-inflammatory cytokines

It is shown that the median serum IL-4 levels in HPV-positive patients with cervical precancerous conditions of low- or medium-avidity IgG antibodies to HSV-1 and/or HSV-2 was respectively 3.6 pg/ml (0 to 6.8) and 3.2 pg/ml (5 to 0.1). In the control median serum, IL-4 levels were 0.1 pg/ml (0 to 0.305). Although we observed a trend towards increased IL-4 in the serum of patients relative to controls, the difference between all these parameters was not statistical.

The median serum IL-10 levels in HPV-infected patients with low- or medium-avidity IgG antibodies to HSV-1 and/or HSV-2 was respectively 7.6 pg/ml (0.1 to 44.8) and 2.2 pg/ml (0 to 50.3). In the control median serum, IL-10 levels in serum was 11.2 pg/ml (2.5 to 36.9). The difference between all these parameters was also not statistically significant.

There was no statistical difference between the levels of IL-4 in the serum of HPV-negative patients with cervical precancerous conditions of low- or medium-avidity IgG antibodies to HSV and that in the control. The median serum IL-4 levels in those patients with low- or medium-avidity IgG antibodies to HSV-1 and/or HSV-2 was respectively 1.8 pg/ml (0 to 16.0) and 1.0 pg/ml (0 to 6.4).

In HPV-negative patients with low- and medium-avidity IgG antibodies to HSV, the median serum IL-4 levels were respectively 253.8 pg/ml (62.1 to 533.9) and 31.0 pg/ml (7.2 to 52. 1). The difference between these figures is statistically significant (*p* < 0.05). The level of serum IL-10 in HPV-negative patients with low-avidity IgG antibodies to HSV-1 and/or HSV-2 was also statistically higher than the level of this cytokine in serum HPV-infected patients both with low- and medium-avidity IgG antibodies to HSV-1 and/or HSV-2 as well as in the control (*p* < 0.05).

**Figure 6:**
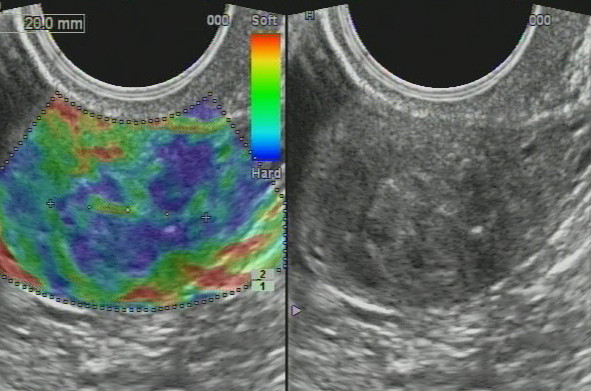
**Changing the amount of TGF-β1 in serum of patients with the cervical precancerous diseases.** In cervical specimen of HPV DNA with low- and medium-avidity and IgG antibodies to HSV-1 and/or HSV-2 (data are presented as M ± Std. Dev). 1, control group (clinically healthy women); 2, patients with low-avidity IgG antibodies to HSV-1 and/or HSV-2; 3, patients with medium-avidity IgG antibodies to HSV-1 and/or HSV-2.

However, the contents of another anti-inflammatory cytokine TGF-β1 in serum significantly increased in all patients with the cervical precancerous conditions as low- and medium-avidity IgG antibodies to HSV-1 and/or HSV-2 compared with those in the control group (*p* < 0.001) (Figure 6).

Thus, we found no correlation between the changes in serum IL-4 and the presence of these two groups of patients compared with precancerous diseases with low- or medium-avidity IgG antibodies to HSV-1 and/or HSV-2 in serum. The level of serum IL-10 increased only in HPV-negative patients with low-avidity IgG antibodies to HSV-1 and/or HSV-2. However, the level of TGF-β significantly increased in the serum of patients of all groups compared.

### Imaging findings - potential ultrasound biomarkers

Histologic examination of the cervical specimen in the first and second groups showed CIN grade I in 31 cases, CIN grade II in 28 and CIN grade III in 22 patients. We have not registered specific differences between the first (HPV-positive) and second (HPV-negative) groups.

In patients of both first and second groups, we registered the changes of structure of the cervix on ultrasound as follows: cervical canal thickening over 5 mm (28 patients, 39.4%); hydrocerix, fluid in cervical canal in ovulatory phase (23 patients, 32.39%); nabothian cysts in cervix (30 patients, 42.3%); local stiff cervical lesions, fibrosis (hyperechoic bands) in cervical tissue (29 patients, 40.8%); deformation of structure, rough boundary between the mucosa and muscle layer (27 patients, 38%); cervicosis, including stiff areas on sonoelastography of hyperechoic inclusions (fibrosis/petrifications) (25 patients, 35.2%); cervical canal polyps (14 patients, 19.7%); increased vascularization in endocervix (22 patients, 31%); extensive fibrosis (hyperechoic bands) in cervical tissue (7 patients, 9.9%); cervical solid nodules; stiff in sonoelastography (3 patients, 4.2%) and increased vascularization in endocervix and stroma (6 patients, 8.45%). At sonography, mean cervical length before treatment was 26.7 ± 6.9 mm and 21.2 ± 4.5 in controls (*P* > 0.05).

**Figure 7:**
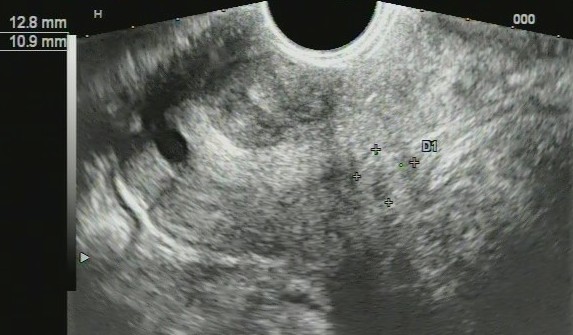
**Ultrasound scans of cervical dysplasia. (A)** Mild cervical dysplasia. **(B)** Severe cervical dysplasia. **(C)** Sonoelastography reveals stiff areas in severe dysplasia. **(D)** Cervical nodule. **(E)** Sonoelastography reveals stiff nodule in severe dysplasia.

For all US symptoms inherent to *severe* and *moderate cervical dysplasia* (CIN grade II, III) (Figure 7), we obtained statistical significance comparing to control group (*P* < 0.01); for *mild cervical dysplasia* (CIN grade I), data were insignificant (*P* > 0.05) due to small number of patients.

Diagnostic evaluation of ultrasound for revealing cervical dysplasia and staging was as follows: the sensitivity was 97.18% (95% CI, 90.17% to 99.58%); specificity was 83.33% (95% CI, 65.27% to 94.30%); positive predictive value was 93.24% (95% CI, 84.92% to 97.74%) and negative predictive value was 92.59% (95% CI, 75.67% to 98.88%).

The ultrasound findings are presented on Figure 7; the distribution of US biomarkers for CIN grades is presented on the Table 2. 

**Table 2:** The ultrasound biomarkers in patients of three groups

**US sign**	**Cervical dysplasia (*n*** **= 71)**	**Controls (*n*** **= 30)**
Mean cervical length, mm	24.8 ± 6.2	21.2 ± 4.5
Mild cervical dysplasia (CIN grade I, *n* = 31)		
Cervical canal thickening over 5 mm	28	6
Hydrocerix - fluid of cervical canal is ovulatory phase	23	7
Nabothian cysts in cervix	30	14
Local stiff cervical lesions, fibrosis (hyperechoic bands) in cervical tissue	29	12
Moderate cervical dysplasia (CIN grade II, *n* = 28)		
Deformation of structure, rough boundary between the mucosa and muscle layer	27	6
Cervicosis stiff in sonoelastography areas of hyperechoic inclusions (fibrosis/petrifications)	25	-
Cervical canal polyps	14	-
Increased vascularization in endocervix	22	5
Severe cervical dysplasia (CIN grade III, *n* = 18)		
Extensive fibrosis (hyperechoic bands) in cervical tissue	7	-
Cervical solid nodules; stiff in sonoelastography	3	-
Increased vascularization in endocervix and stroma	6	-

## Discussion

Today, it is known that in the development of HPV-induced cervical dysplastic lesions, the key impact has a specific cellular immune reactivity to HPV, especially the Th1-type response of T helper cells, that develops by balancing various opposition groups of cytokine, in particular, pro- and anti-inflammatory cytokines [9,14,35,36]. This is confirmed by the fact that patients with immunodeficiency states with suppressed cellular immunity, such as AIDS, are extremely susceptible to HPV-induced cancers [8,9].

It is known that suppression influenced oncoproteins E6 and E7 of HPV high-risk oncogenic gene expression of interferon, interferon genes and IL-18 production, which directly regulates the γ-interferonogenesis, as well as increased production of immunosuppressive cytokines that inhibit the development of T helper cells of Th1-type and the production of interferon-γ and IL-2, which is one of the most important evolutionary mechanisms against HPV immune factors [9,18,19].

It is believed that another important co-factor in HPV-induced neoplastic lesions is HSV infection [8,25-30,37]. Therefore, we determined whether there is a connection between the simultaneous infection of HPV and HSV patients and changes in production of pro- and anti-inflammatory cytokine ratio analysis which allows to describe the direction of the immune response toward predominant formation of cellular (Th1 pathway) or humoral (Th2- pathway) reactions.

Assessment of cytokine production was performed by determining their concentration in serum, since such studies are widely used in laboratory practice to diagnose the state of immunity. Therefore, *we hypothesized* that the results of our study may have important *diagnostic* and *predictive* clinical value, contributing to the *predictive diagnosis* of herpetic infections in HPV-induced cervical precancerous diseases, determining besides the presence of antibodies to HSV-1 and HSV-2 in serum, the avidity of specific IgG antibodies to these herpes virus. Determination of avidity of specific IgG is important for the diagnosis of various stages of HSV infection and its development is characterized by the presence of antibodies with different (low, medium or high) avidity. We have previously shown [30] that in the serum of patients with cervical precancerous conditions, medium-avidity antibodies to HSV-1 and/or HSV-2 were generally low.

In this study, we have shown that there is a relationship between infection patients with cervical precancerous conditions HPV and/or HSV-1 and HSV-2 suppression γ-interferonogenesis. The important role is played by the degree of avidity of IgG antibodies to HSV-1 and/or HSV-2. In HPV-infected patients with low-avidity IgG antibodies to HSV-1 and/or HSV-2, level of IFN-γ in serum was significantly lower than in patients with medium-avidity IgG antibodies to these herpes virus and in controls. HPV-infected patients with low-avidity IgG antibodies to HSV-1 and/or HSV-2 often showed HPV oncogenic high and medium risk oncoproteins E6 and E7 which are known to suppress γ-interferonogenesis [9,18,19].

Previously, we have reported that in severe HPV-induced cervical precancerous diseases, as CIN-II and CIN III, it was observed that there is a more common incidence of HPV-positive patients with low-avidity IgG antibodies to HSV-1 and/or HSV-2 compared to the patients whose blood serum contained medium-avidity IgG antibodies to HSV-1 and/or HSV-2 [30]. In patients with CIN III, cancer *in situ*, the production of IFN-γ was inhibited more clearly than in CIN I and in benign cervical processes [15].

Whereas, among patients with cervical precancerous diseases without herpetic infection in clinical form, the IgM antibodies to HSV-1 and/or HSV-2 [30] were absent in their serum; it is likely that the presence of low- and medium-avidity IgG antibodies to HSV-1 and/or HSV-2 shows the final stage of primary HSV infection or exacerbation of a chronic process. Production of IFN-γ was inhibited also in HPV-negative patients with cervical precancerous diseases of low- and medium-avidity IgG antibodies to HSV-1 and/or HSV-2. Thus, the suppression of γ-interferonogenesis may be due to both HPV infection as HSV-1 and/or HSV-2.

Still, it is not clear whether infected patients with high- or medium-risk HPV types lead to reactivation of HSV-1 or HSV-2 genome in the case of chronic process.

The analysis of our data may argue that HPV and HSV-1 or HSV-2 suppressing of γ-interferonogenesis may contribute to the development of HPV-induced cervical cancer, since this cytokine is known [9] to control the progress and growth of HPV-induced tumors.

We have previously shown that the HPV induced cervical dysplasia in patients with decreased serum levels of IFN-γ and IFN-α while increasing the levels of pro-inflammatory cytokines, TNF-α and IL-1β, and cytokine Th-2 type, IL-4 [9,15]. According to other authors in HPV-induced warts, in the peripheral blood of patients, there is also a decreased level of IFN-γ; the level of TNF-α, IL-4 and IL-10 was significantly increased compared with that of the controls [38].

In patients with cervical precancerous diseases with different avidity IgG antibodies to HSV-1 and/or HSV-2, we have not observed the development of an active inflammatory response, as the content of pro-inflammatory cytokines like IFN-α, IL-1β, IL-2 and TNF-α maintained at level of controls.

However, in HPV-infected and HPV-negative patients with cervical precancerous disease with low- or medium-avidity antibodies to HSV-1 or HSV-2, the concentration of serum TGF-β 1 was significantly increased. It was reported [21] that TGF-β1, contributing to chromosomal aberrations of HPV-infected cervical epithelial cells, plays an important role in the early stages of cervical carcinogenesis. However, HPV-transformed cells themselves produce TGF-β1 and certain other immunosuppressive cytokines, such as IL-10 [20]. TGF-β1 is a pleiotropic cytokine that can both inhibit and stimulate cell proliferation, but tumor cells often lose their sensitivity for cytokines [39]. Most researchers still link the elevation of TGF-β1 production with increased tumor growth [40-42]. It was found that the progression of cervical intraepithelial neoplasia from mild to severe stage correlates with increased TGF-β1 gene expression in cervical samples (with smear scrape by Papanicolaou) [40].

Therefore, the increase of TGF-β1 production was registered in the present study in all compared groups, which is probably the result of precancerous processes of cervix than the result of HPV infection and/or HSV-1 and HSV-2 and therefore should be considered as unfavorable predictive sign for these diseases.

Thus, our findings showed that HPV-positive patients with low-avidity IgG antibodies to HSV-1 and/or HSV-2 as well as HPV-negative patients with low- and medium-avidity IgG antibodies to HSV-1 and/or HSV-2 showed the violation of production of IFN-γ, confirming the immunosuppressive state. However, the level of TGF-β significantly increased in the serum of HPV-positive as well as HPV-negative patients of all groups.

While for HPV-negative patients with low-avidity IgG antibodies to HSV-1 and/or HSV-2, the production of IL-10 increased. The level of IL-10 in serum also increased in HPV-positive patients with CIN III according to other studies [16].

According to the literature data, the reduction of IFN-γ and IL-2 and increased levels of IL-4 and IL-10 in serum may be considered as a *predictive biomarker* for unfavorable prognosis in some forms of HPV-induced cancers as a shift from Th-1 to Th-2 cytokines observed in metastases and in later stages of carcinogenesis [16,43].

Therefore, the presence of low-avidity IgG antibodies to HSV-1 and/or HSV-2 in the serum of patients with HPV-induced cervix precancerous diseases does not depend on the reactivation of HSV genome (genital herpes or generalized herpetic infection) which may be an indication for treatment as immunomodulators and antiviral (antiherpetic) drugs.

### Expert recommendations

HPV-positive patients with low-avidity IgG antibodies to HSV-1 and/or HSV-2 in the serum showed the violation of production of IFN-γ, confirming the immunosuppressive state. Therefore, the presence of low-avidity IgG antibodies to HSV-1 and/or HSV-2 in the serum of patients with HPV-induced cervix precancerous diseases does not depend on the reactivation of HSV genome (meaning it is genital herpes or herpetic system process) which may be an indication for treatment as immunomodulators and antiviral (antiherpetic) drugs. The level of TGF-β significantly increased in the serum of HPV-positive as well as HPV-negative patients with low- and medium-avidity IgG antibodies to HSV-1 and/or HSV-2. Ultrasound was used for the diagnosis of cervical dysplasia, and it showed the sensitivity 97.18%, specificity 83.33%, positive predictive value 93.24%, and negative predictive value 92.59%.

### Future outlooks and recommendations

Considering the limitations of the current research, more studies on larger cohorts using extensive technological platform with the translational research approach are required for the assessment of different constitutional types, collateral diseases, etc. with biomarker specificity/sensitivity calculations to formulate valid panel aiming for implementation for PPPM in complex women health. We suggest also the further studies regarding virus infections, human microbiota, immune response and gene associations and interactions with promising substances in nanoscale (e.g., nanoceria, nanogold) for complex impact for female health considering wide spectrum of comorbidities and related pathologies to initiate comparative studies to establish science-based treatment algorithms and update screening programs. After approval, develop safe and effective person-related treatments beneficial for individual outcomes.

### PPPM approach in complex women health

Integrative women health care includes multimodal approach for gynaecologic pathology management with PPPM paradigm including the assessment in the following: breast [3], endometrial [4] precancers, etc. and collateral diseases as endocrine, autoimmunity, neurological, including neurodegenerative, vaccine-related disorders, pelvic pain management [44] in a point of view of predictive diagnosis for personalized treatment and tailored preventive measures.

### Cancerogenesis

It was acknowledged that more than 15% of viral infections are able to cause cancer in humans [45,46]. Thus, HPV infection is attributed to 80% of all human cancers and was supposed to play a central role in the development of breast cancer [47-49]. Thus, among persistent HPV infection, one of about 15 genotypes of carcinogenic human papillomavirus causes almost all cases [50].

Schiffman et al. [50] described four major steps in cervical cancer development:

1. Infection of metaplastic epithelium at the cervical transformation zone, viral persistence, progression of persistently infected epithelium to cervical precancer and invasion through the basement membrane of the epithelium;

2. Infection is extremely common in young women in their first decade of sexual activity;

3. Persistent infections and precancer, typically within 5 to 10 years, from less than 10% of new infections;

4. And finally, the invasive cancer that arises over many years, even decades, in a minority of women with precancer, with a peak or plateau in risk at about 35 to 55 years of age.

Each genotype of HPV acts as an independent infection, with differing carcinogenic risks linked to evolutionary species.

### Obesity, diabetes, diet and lifestyle-related issues

Metabolic disturbances in obesity causes a number of diseases, namely cardiovascular system, number of tumor sites, including lung cancer, breast cancer, uterine and ovarian cancer; in women, there is a violation of ovarian-menstrual cycle, local and systemic immunity and dyslipidemia. The recent data, regarding women’s differential responses to lifestyle changes, support another branch of research with a gender nutrition emphasis within predictive, preventive, and personalized medicine [51]. There were illustrated extensive interrelations among virus action, cellular oxidative stress, gene damage, multiple immune pathways and proteomic changes in diabetes mellitus, cancer and many chronic disorders development, many of them were also related to HPV infection [52].

### Expand the immunologic study

In immunology, the signal pathways are still not studied for gynecology cancers, promising is the ability of group of factors considered, namely, probiotics, which affect the relevant Toll-like receptors (TLRs) that promote effective immune response and the initiation of an effective immune defence. Application of probiotics/immunobiotics might be a promising and integrative personalized use.

### Oxidative stress

*Oxidative stress* (OS) induced by reactive oxygen species (ROS) is one of the main factors in cellular aging, and many cellular disorders cause extensive damage to DNA and also in mitochondria [45]. OS is an interesting promoting factor in HPV-initiated carcinogenesis; however, its role has received little attention in this regard. In inflammation, ROS and nitric oxide (NO), generated by inflammatory cells, play a key role in carcinogenesis. Thus, ROS can induce the formation of 8-oxodG, an indicator of oxidative DNA damage while NO can induce the formation of 8-nitroguanine, a marker of nitrative DNA damage. These factors are potentially mutagenic, which may account for the cancer-promoting effect of inflammation. It is reported that high-risk HPV types promote inducible nitric oxide synthase-dependent DNA damage, which leads to dysplastic changes and carcinogenesis [45]. While therapeutic treatments cannot be based exclusively on the abatement of oxidative stress, neutralizing this cellular disorder could minimize collateral damages associated with the transformation of biomolecules in the cytosol.

### Nanotechnologies - the challenge for advanced diagnosis, treatment and prevention

Advances in nanoscience, nanotechnology and nanomedicine lead to the construction of new materials and devices for various scientific and therapeutic purposes, which are applicable in molecular diagnostics, nanodiagnostics, and improvements in the discovery, design and delivery of drugs, including nanopharmaceuticals. The application of nanoparticles allowing the combination of therapy and diagnosis, known as *theranostic*, has received increasing attention in biomedicine [53]. Pharmacological, pharmaceutical and toxicological aspects of the application of nanoparticles in biomedical purposes still remain poorly understood. While *oxidative stress* has been postulated as one of the main physiopathological hallmarks of most of chronic diseases, the *nanoparticles* of gold [53,54] and cerium dioxide [55] were reported as strong agents against oxidative damage, having anti-aging activity. Nanoparticles of *cerium dioxide* considering its UV-shielding effect, antiviral, antibacterial and antifungal activity, cardioprotective, neurotrophic, hepato- and nephroprotective and anti-aging effect have potential for various biomedical applications.

**Figure 8:**
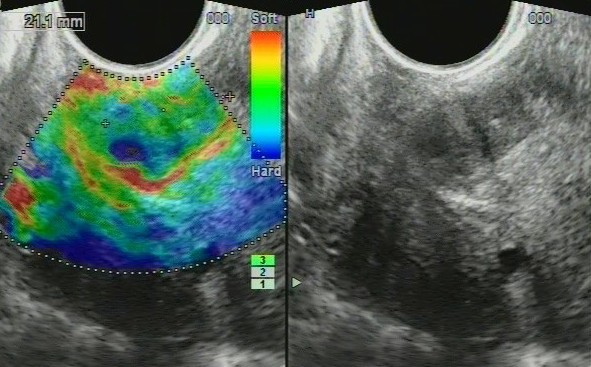
**Alleged scheme of antiviral activity of nanocrystalline cerium dioxide against *the herpes virus.*** *CDN, cerium dioxide nanoparticles.*

The mechanisms of *antiviral activity* of nanocrystalline cerium dioxide were reported [53] as a *universal nature* action and can be directed to different targets in a cycle of virus reproduction (Figure 8). The nanoceria are able to hydrolyze ether-phosphate connections in biological molecules and inhibit phosphorylation of IκB, reducing the activity of NF-κB upon viral replication. Thus, HSV activates IKK-kinase, while phosphorylation of the inhibitory protein IκB causes transcriptional activation of nuclear factor NF-κB; in turn, NF-κB activation increases the expression of the viral gene. Development and introduction into clinical practice of drugs that inhibit phosphorylation IκB will open a new direction in the treatment of herpes infections (Figure 8).

If the virus is located near the nanoparticle with similar/smaller size than virus, the interaction between the virus and particle may occur due to fluctuating fields (the analogue of van der Waals forces). Due to fluctuations of the electromagnetic field, interaction between the nanoparticles with sufficiently large values of the non-linear polar leads to capacity building, which causes attraction at large distances, and at distances of the order of particle sizes - repulsion.

The antiviral activity mechanisms of nanoceria that we discovered are of particular interest and are for further study because these open the prospect of using biosafe and biocompatible nanoconstructions to perform beneficial prevention and treatment of viral diseases for patients.

Treatment with nanoceria has supplementary perspectives in gynecology and reproductive medicine as it results in the increase in the number of oocytes in follicles, increase in the number of oocytes at metaphase I and metaphase II, increase in the number of living granulosa cells and decrease in the number of necrotic and apoptotic cells [55].

Developing the technological platform for *synthesis of virus-like nanoparticles* is promising for designing safe and effective personalized vaccines, and that in combination with anticancer theranostic application, it is a significant impact in order to develop in PPPM in women health and reproductive medicine. *Nanosensors* could be used for screening the affinity between the identified proteins and the immunological synapses; protein arrays can be used to discover new antigenic determinants for vaccine development.

### HPV vaccines

*Vaccines* offer a safe and cost-effective *prevention*; on the other hand, disappointing results of vaccine-related disorders require the shift of the ‘risk curve’ in order to better select cheap, efficacious and well-tolerated vaccine candidates. Vaccines do not eliminate the risk of HPV-induced cervical cancer; cervical screening programs will still be required to minimize cancer incidence. Thus, to develop innovative HPV vaccines and in order for the vaccines to reach populations in greatest need, financing mechanisms and multidisciplinary partnerships is essential [56,57]. The administration of HPV vaccine to HPV-naive women (in schools), as well as to women who are already sexually active to reduce the incidence of HPV16/18-related cervical precancers and cervical cancer [57] is discussed. The development of new successful *personalized* treatment, prospective to promote effective immune response to infectious and related diseases, depends on the early detection of the etiological agent and any antibiotic resistances in a point of view of individual predisposition.

### Mathematical models

We suggest to follow up this study in regards to profound assessment of all the panel of biomarker information using advanced biostatistics and bioinformatics techniques. We can suggest a mathematical model that integrates those multiparameter data, according to which the medical process is perceived as a complex system like ‘black box’ [58,59] and can describe the process (in particular, HPV-induced cancerogenesis) by some of the primary indicators (panel of biomarkers). So primary indicators and output rate are stochastic in nature and presented as statistical information. Such model may lead to integrate all biomarkers of the panels to be applicable in real PPPM practice in the HPV-induced cervical precancerous lesions.

### Development of biomarkers panel

Several biologic markers or indexes have been studied as potential tools to determine the prognosis and biological behavior of cervical cancer [60]. It was suggested that new *HPV-oriented model* of cervical carcinogenesis should update and gradually replace older morphological models based only on cytology and histology [50]. It can be promising to develop biomarkers, fed through different techniques, that are applicable to minimize the incidence of cervical cancer and the morbidity and mortality it causes even in low-resource settings, leading to improve prevention and clinical management strategies, including improved screening tests and vaccines.

*Phenotypic* (at the tissue, cellular, and molecular levels) and *genotypic* biomarkers are potential surrogate end points for cancer incidence [61]. This would require that both the phenotype and genotype of the target tissue in agent-treated subjects, especially in any new or remaining precancers, are equivalent to or show less progression than those of the placebo-treated subjects.

Precancer (intraepithelial neoplasia) has been considered the primary *phenotypic* surrogate end point. Promising are the biomarkers measuring specific and general *genotypic* changes correlating to the carcinogenesis (e.g., progressive genomic instability as measured by loss of heterozygosity or amplification at a specific microsatellite loci). Thus, virus induce the activation of c-MYC protein suppressing the cell cycle controlling activity of P53 and allows, therefore, the development of new tumorigenic *phenotype* of transformed human cells. In consensus, the activated synthesis of HPV proteins E6, E7, E1 and E2 has been shown to be involved in the induction of malignant cell transformation [45,48], a key interaction as regards to oncoproteins E6 and E7 of HPV types of high oncogenic risk of intracellular factors that play an important role in the regulation of growth, differentiation, and apoptosis.

Integration of HPV DNA of high oncogenic risk to the host cell genome is undoubtedly a major factor in the persistence of the virus and its carcinogenic potential. However, there was an obtained evidence of other risk factors for HPVI and malignant transformation of HPV-infected cells [10]. In particular, the important role of the immune defence of the organism, especially cellular immunity and cytokine production of Th1-type IFN-γ and IL-2, is to execute control over the viral infection and tumor growth [12]. HPV oncoproteins can evade immune surveillance of the factors or even cause immunosuppression by manipulating the immune mechanisms of the host cell. In addition, the recently established oncoproteins E6 and E7 of HPV types of high oncogenic risk inhibit gene expression of interferon and interferon-induced genes and reduce the sensitivity of cells to IFN. This is one of the most important mechanisms of carcinogenic action [18] because the development and nature of the viral diseases depend on the specific interaction of the virus-cell system where its leading role has different types of interferon. *Therefore, violation of interferon production may underlie relapse HPVI and be a risk factor for the development of HPV-induced malignant tumors.*

*Biomarkers of immunosuppression may be considered as follows*[8,9,38]: the levels of *various cytokines*, namely IFN-γ, IL-2 and IL-12; cytotoxic T-lymphocyte antigen 4 (CD4), glucocorticoid-induced tumor necrosis factor receptor (TNFR)-related protein and programd cell death protein 1; natural killer (NK) cell biomarkers (receptors located on the surface of NK), namely levels of NKG2D and NKp46 and the expression levels of tumor necrosis factor-α, IL-4 and IL-10. Increased expression of MyD88 and TLRs is likely to enhance immunosuppression of Tregs, leading to the imbalance of Th1/Th2 and cytotoxic T cell type 1 (Tc1)/Tc2 cells. Our results illustrated that the presence of low-avidity IgG antibodies to HSV-1 and/or HSV-2 is the biomarker of *immunosuppressive state.*

Other potential surrogate end points that may occur earlier in carcinogenesis may include *proliferation and differentiation indices*, specific gene and general chromosome damage, cell growth regulatory molecules and biochemical activities (e.g., enzyme inhibition); Ki67, a nuclear proliferation associated antigen is expressed in the growth and synthesis phases of the cell cycle (G1, S, G2 and mitosis). Serum biomarkers also may be monitored because of their accessibility [61].

The expression patterns of three potential dysplastic biomarkers, p16^INK4^A, CDC6, and MCM5, was discussed and compared in [62] to evaluate their use as predictive biomarkers in squamous and glandular cervical preinvasive neoplasia. Combinations of biomarkers may be useful in difficult diagnostic cases. Thus, *p16*^*INK4A*^ expression is closely associated with high-risk HPV infection; *MCM5* staining intensity is independent of high-risk HPV infection, highlighting its potential as a biomarker in both HPV dependent and independent cervical dysplasia, and *CDC6* may be a biomarker of high-grade and invasive lesions of the cervix, with limited use in low-grade dysplasia. Thus, p16^INK4A^ may be suggested as the most reliable marker of cervical dysplasia among dysplastic group [62].

Current data support the association of *oral contraceptive* use with cervical adenocarcinomas *in situ*; however, no other evidence was found that oral contraceptives independently increase the risk of cervical carcinomas [63].

The *insulin-like growth factor (IGF)* system is organized in a complex regulatory network at the cellular and subcellular levels. The IGF system has a key physiological role in the development of the organism and maintenance of normal cellular function during fetal and postnatal life and plays a central role in many aspects of the development and progression of cervical cancer. The IGF system consists of three ligands, IGF-I, IGF-II and insulin; three cell membrane receptors, IGF-I receptor (IGF-IR), insulin receptor (IR) and IGF-II receptor (IGF-IIR); and six high-affinity IGF binding proteins, IGFBP − 1 through −6, their specific proteases (IGFBP proteases) and membrane receptors (IGFBP-R). IGF-I is a potent mitogenic growth factor that plays a critical role in cancergenesis [64].

#### Hormones

Immunohistochemistry (IHC) is probably the most affordable and simple technology to detect many biomarkers [60]. The differences in immunohistochemical expression of p53, bcl-2, bax, estrogen receptor (ER), and progesterone receptor (PR), androgen receptor (AR), progesterone receptor antagonists (PA), etc. should be properly assessed to find *the most common diagnostic pitfalls and helpful morphologic and immunohistochemical markers*[4]*.*

#### Oncomarkers

Elevated CA 125 levels are detectable in 20% to 75% of patients with cervical adenocarcinoma and have been associated with advanced tumor stage, large tumor size, high histological grade, lymph node involvement and status [64].

Vieira et al. [65] proposed anti-CD34 as a marker for evaluating angiogenesis in cervical cancer [66]. Anti-CD34 antibody is a highly sensitive marker for endothelial cell differentiation and has also been studied as a marker for vascular tumors [67]. A recent study supports the power of serum markers such as squamous cell carcinoma antigen (SCC), CYFRA 21–1, CA 125, immunosuppressive acidic protein (IAP) and vascular endothelial growth factor (VEGF) in patients with cervical cancer [65].

### Proteomics

Proteomics is a powerful tool for the development of molecular biomarkers in the postgenomic era and provides the hope of discovering novel biological markers for use in the screening, early diagnosis and prediction of response to therapy [68]. Proteomics strategies are powerful enough to identify novel co-carcinogenic factors and to understand the mechanisms of tumor development, interplay between viral infection and protein dysfunction, considering selective interaction of viral oncogenes with a subset of intracellular proteins mainly involved in apoptosis resistance, cell growth and differentiation and cell transformation [69].

Proteomic studies of membrane proteins are an analytical challenge due to their dynamic physicochemical characteristics, hydrophobicity and heterogeneity. Differences in the membrane proteomes of two cervical cancer cell lines may correlate with the invasive potential [70]. The results of studies on membrane proteomes of cervical cancer cell lines, differing in viral status and invasive phenotypes are the following: HeLa, an invasive HPV positive cell line, and C33-A, a non-invasive HPV negative cell line [70]. Metastasis is the most common cause of mortality in cervical cancer. Key molecular elements of the plasma membrane and cytoskeleton are responsible for cell motility and invasion.

Human papillomavirus oncogenic proteins E6 and E7 were suggested to target the p53 and Rb pathways; E6 can enhance telomerase activity, whereas E7 inhibits a p16*ink4A*-dependent pathway that limits cellular proliferation in epithelial cells [71]. Thus, HPV oncogenes induce genomic instability and allow the cells to acquire accumulating genomic alteration, thus ultimately leading to the full neoplastic state. HPV E6 and E7 oncoproteins allow for the accumulation of genetic mutations and the survival of mutated cells, their expression also contributes to the immortalization of infected cells [72]. Only a minor percentage of viral infections lead to invasive growth, thereby indicating insufficient role of viral oncogene expression that could be either related to a long-term viral protein expression or the results of many co-factor(s), namely, viral and non-viral; host, and environmental factors should be investigated [6,7,73], including the role of pro- and anti-inflammatory cytokines presented in the current study.

Cervical mucous or *cervical vaginal fluid (CVF)* is potentially an ideal sample to screen for biomarkers for early detection of cervical cancer. A recent study identified 151 new proteins that included proteins present in the lower female genital tract, such as HBD-2 and cathelicidin, two proteins that play an important role in the innate cervicovaginal immunity [74].

### Imaging (US, MRI, PET-CT)

Magnetic resonance imaging (MRI) is the most effective technique to assess the type, degree of differentiation, presence or absence of lymphovascular invasion, lymph node involvement, etc. Magnetic resonance spectroscopy (MRS) may support additional characteristics, namely ADC and total choline, that may be suggested in a role of predictive biomarkers. Thus, ADC coefficients were reported to be lower in cancer compared to normal cervical tissue, with degree of tumor differentiation contributing to this difference [75]. Baseline ADC and focal regions of ADC restriction predict for partial response with moderate sensitivity and specificity in patients with postoperative recurrences of cervical cancer and need to be validated in larger cohort. Chopra et al. investigated diffusion-weighted MR imaging (DWI) as a response biomarker in patients undergoing chemoradiation for postoperative recurrences of cervical cancer [76].

*Positron emission tomography-computed tomography (PET-CT)* has higher sensitivity and specificity than do conventional anatomic modalities and is valuable in determining the extent of disease and detecting recurrent or residual tumor [77]. In locally advanced cervical cancer, ^18^F-fluorodeoxyglucose (FDG) positron emission tomography-computed tomography (PET/CT) has become important in the initial evaluation of disease extent. ^64^Cu-labelled diacetyl-di(N(4)-methylthiosemicarbazone) is taken up by hypoxic tissues, which may be valuable for prognostication and radiation treatment planning [78]. However, Schöder et al. discuss the potential financial, legal and radiation safety implications associated with using whole-body PET/CT for cancer screening, diagnosing, staging and restaging cancer and for monitoring treatment effects. In spite of advocating CT, PET or PET/CT for whole-body screening, recommendations and decisions regarding cancer screening should be based on reliable data, not good intention, assumptions or speculation [74]. *For these reasons, actually, we still cannot consider PET as a perspective screening tool for cervical cancer.*

#### Elastography - promising non-invasive biomarker

Schöder and Gönen [79] investigated elastography of the uterine cervix in non-pregnant women and showed that there was no correlation between cervical stiffness and patient age, but Thomas et al. found that it was possible to diagnose malignant tumors of the cervix using this method [80]. Authors hypothesize that the directionality of the tissue mechanical response is primarily due to preferential collagen orientation in the cervical stroma, suggesting that cervical tissue is mechanically anisotropic with a uniaxial response dependent on the direction of loading, the anatomical site of the specimen and the obstetric history of the patient [80]. Differences between the soft internal os and harder external parts of the cervix are good predictors of a favorable reaction to oxytocin during induction of labor [81].

*Dopplerography* of the cervix revealed significant differences in all existing indices studied between women with cervical precancerous lesions or cancer and healthy women. In women with cervical cancer, an advanced stage is associated with higher velocity indices [82,83]. High vascularity has been known as a characteristic of grade 3 CIN and invasive lesions; angiogenesis has been associated as indicator of prognosis. There is a study demonstrating that microvessel density in carcinomas of the uterine cervix is a factor associated with poor prognosis [84]. Pelvic congestion and vascular redistribution are involved to the pathogenesis of being a promising supporter for imaging and lab biomarkers. Peripheral microcirculation assessment might be considered to support a supplementary information for cervical cancer patients [85]. *Laboratory biomarkers representing vascularization may be conjoined with imaging data*[66,67]*in particular for vasospasm assessment.*

The most specific colposcopic signs of PVI are acetowhite epithelium, positive iodine punctuation and mosaic, and atypical transformation zone [9].

*Numeral challenges for optical imaging optimization* were suggested, e.g., enhancing techniques with use of quantum dots that provide a promising alternative to conventional organic dyes for biological imaging, that when combined with optical imaging technologies can help visualize malignant changes in cervix at the molecular level [86].

Thus, here, we suggest the *panel of biomarkers* (already proved and potential) for cervical carcinogenesis based on the currently available HPV-oriented model, leading to improve prevention and clinical management strategies, including improved screening tests and vaccines, reliably diagnosing that can help in the choice of multiple therapeutic alternatives most likely to benefit the patients [66], and to minimize the incidence of cervical cancer and its morbidity and mortality, even in low-cost settings.

### Panel of biomarkers

The following are the panel of biomarkers:

1. Phenotypic (at the tissue, cellular, and molecular levels)

• *Proteomic* biomarkers (proteins of enzymes, growth factors, cell adhesion molecules, calcium-binding proteins, proteases, protease inhibitors, transporter proteins, structural molecules, apoptosis inhibitor, molecular chaperone, as well as proteins related to cell growth, cell differentiation, cell transformation, tumor invasion, carcinogen metabolism, etc.) [87];

• Serum biomarkers of *proliferation and differentiation* (growth regulatory molecules, and biochemical activities (e.g., enzyme inhibition); Ki67, a nuclear proliferation associated antigen);

• The *histological biomarkers* of cervical HPVI: exophytic and typical flat warts, mild changes of plane and metaplastic epithelium (single koilocytes) condylomatosis cervicitis, CIN of varying grades with/without koilocytes, and *cervical cancer*[9];

• *Dysplastic* biomarkers: p16INK4A, CDC6 and MCM5 [56];

• *Viral* biomarkers: virus herpes, HPV-related biomarkers (viral proteins E6, E7, E1 and E2, etc.);

• *Receptors* (ER) and PR, AR, PA);

• *The insulin-like growth factor (IGF) system* receptors (IR, IGF-IR, IGF-IR/IR hybrids and IGF-IIR), the peptides (IGF-I, IGF-II and insulin), six high affinity IGFBPs (−1 to −6), IGFBP proteases and IGFBP receptors (IGFBP-R);

• *Cancer antigen* (biomolecules) based biomarkers: cancer antigen 125 (CA125);

• *Receptors* of the diffuse neuroendocrine system (amine precursor uptake and decarboxylase, APUD cells);

• *Apoptosis* biomarkers (Ki67, etc.);

• CVF samples for HBD-2 and cathelicidin testing;

• *Oxidative stress* biomarkers: DNA oxidation biomarkers (oxidized DNA bases such as 8-OHdG, autoantibodies to oxidized DNA, modified comet assay), lipid oxidation (thiobarbituric acid-reactive substances, exhaled pentane/ethane, low-density lipoprotein resistance to oxidation, isoprostanes), protein oxidation (protein carbonyls) [45,88];

• *Nitrosative stress* biomarkers: NO, nitrite, peroxynitrite, and inducible NO synthase (iNOS) expression, modificated proteins, nitrosothiols, 8-nitroguanine (a marker of nitrative DNA damage), etc. [45,89,90];

• *Vasospasm* biomarkers (endothelin, NO, etc.) [91,92].

2. Genotypic biomarkers (oncogenic types of HPV, c-MYC protein system, activity of P53, bcl-2, bax, TFPI2, Secreted Protein Acidic and Rich in Cysteine (SPARC) [93,94],etc.).

4. Imaging biomarkers

• *MR imaging/MR sprectroscopy*: metabolic biomarkers, diffusion-weighted imaging, etc.;

• *Ultrasound* (conventional, dopplerography; sonoelastography, performed in specific phases of cycle; nabotian cysts; cervicosis, polyps; hydrocerix; stiff cervical lesions, fibrosis; pelvic congestion, ovulation assessment);

• *PET-CT* (has rather poor screening value);

• *Colposcopy*: specific colposcopic signs of PVI are acetowhite epithelium, positive iodine punctuation and mosaic, atypical transformation zone [9], develop *in vivo* subcellular imaging, enhancing optical imaging techniques as quantum dots [64];

• *Pathology, immunohistochemistry* (immunohistochemical expression of p53, bcl-2, bax, ER, and PR, AR, PA, etc.);

• *Cellular, subcellular imaging*.

4. Immunological biomarkers

• *Biomarkers of immunosuppression*: immunosuppressive acidic protein (IAP); low-avidity IgG antibodies to HSV in HPV-positive patients leading violation of production of IFN-γ; various cytokines in the peripheral blood that IL-2, IL-12 and interferon-γ; cytotoxic T-lymphocyte antigen 4, glucocorticoid-induced TNFR-related protein and programd cell death protein 1 [38]; NK cells biomarkers (levels of NKG2D and NKp46, natural killer (NK) cell activation receptors located on the surface of NK); the expression levels of tumor necrosis factor-α, IL-4 and IL-10; expression of MyD88 and TLRs is likely to enhance immunosuppression of Tregs, leading to the imbalance of Th1/Th2, cytotoxic T cell type 1 (Tc1)/Tc2 cells.

5. Biomarkers in pregnant women [95];

6. Patients profile including history of collateral diseases (cancer, diabetes, infections, etc.), HPV infection and sexual history data from properly constructed questionnaires, and cytological screening [57].

### Education for preventive measures

Educational programs and individual preventive facilities for sexually active persons are important tasks for PPPM. The material for dissemination and lecturing should be standardized (well translated, to be easily understood) in order to facilitate the work. Disseminate information and perform campaign for organization of free health check up available in low cost (ultrasound, cytological screening, most valid biomarkers) and vaccination in childhood, especially in developing countries, and in time implementation of novel scientific findings in the field. Support preventive educational activity with long-term commitment of private and public funding programs.

### Potential economical benefits of PPPM

*Cancer of the cervix* accounts for over 60% of the gynecological cancer burden in developing countries which causes about 500,000 new cases and 250,000 deaths each year [5], despite being preventable by current technologies. The portions of the total costs of cancer have been estimated to be as high as US$895 billion worldwide [96]. The cost of new gynecological cancers in developing countries in 2009 totalled US$1,087 million compared to the US$11.9 billion spent in developed countries; in 2009, cancer burden in EU were estimated as 126 billion €, in particular for cervix uteri which is 2,664 million € [97].

The impact of HPV is not just clinical. The evaluation and treatment of the clinical manifestations of HPV also have an enormous monetary impact on the health care system [98]. The economic burden that was associated with non-cervical HPV-6, 11, 16, and 18-related conditions in the US population in the year 2003 approximates US$418 million (range, US$160 million to US$1.6 billion) [99]. Thus, HPV testing requires more high-technology laboratory-based molecular analyses, involving higher costs. A primary HPV detection test for a routine screening with a higher sensitivity and negative predictive value for the detection of preinvasive disease than cytology, and being in low cost is called to be developed [96]. Many HPV-related diseases namely diabetes and cancer might be considered for integrative preventions within virus, immunology and genetics chain that should benefit the indirect economical effects [100].

### Consolidation of the PPPM concept

Thus, our analysis of assessment of biomarker panel including current research of pro- and anti-inflammatory cytokines in patients with papillomavirus and herpes simplex virus infections allows to conclude as follows:

### Personalized medical approach

Each individual pathological pattern of the production of pro- and anti-inflammatory cytokines in HPV-induced cervical precancerous diseases in patients infected with HSV1 and/or HSV2 in serum IgG, identified with varying degrees of avidity, specific to these herpes virus are promising biomarkers that defines further personalized tactics of comprehensive treatment using immunomodulators and antiviral drugs. The combination of diagnostic modalities such as imaging, serum and CVF biomarkers should help in the choice of multiple therapeutic alternatives as currently available integrative treatment (surgery, cryotherapy, radiotherapy, chemotherapy, etc.) that is beneficial to the patients.

### Predictive medical approach

Assessment of extensive biomarkers panel for cervical carcinogenesis based on *HPV-oriented model* should minimize the incidence of cervical cancer, and the morbidity and mortality it causes, even in low-cost settings, leading to improved prevention and clinical management strategies, including improved screening tests and vaccines. Persistent herpes virus, HPV infections have a high-risk for development of a number of collateral/infection-induced chronic diseases, namely cancer, cardiovascular, endocrine, autoimmune, metabolic, neurological diseases, that require research programs to support high levels of scientific and technological development for novel predictive programs. Levels of pro- and anti-inflammatory cytokines in papillomavirus and herpes simplex virus infections in patients have strong predictive value which has potential for development of predictive biomarkers for personalized treatment and tailored prevention of cervical precancerous lesions. Possibly, an imbalance of cytokine production Th1, 2, 3-type and anti-inflammatory cytokines may underlie CC relapse in HPVI and be a risk factor for HPV-induced malignancies. Expand biomarkers panel and proceed in the validation of new biomarkers in the shortest terms.

### Preventive medical approach

Our results may lead to initiate the programs for women at risk to prevent cervical cancer and improve quality of life. In parallel with extensive biomarker panel develop preventive programs adapted for developing countries, namely campaigns for vaccination at schools, suggesting valid and simple diagnostic tests with self sampling and treatment with basic medicines. It is recommended to promote programs for introduction of ambulatory (‘office’) integrative women health care to a new level of efficiency and safety of the method.

With the concluding points, we can formulate the following proposals (expert recommendations):

1. For the European Union (EU): create an international research project to study integrative diagnosis and treatment towards women health improvement with regards to preserve the reproductive function. Perform sufficient evidence studies to determine relationships in virus, genetics, immune pathways, receptor system, to expand biomarkers panel that will allow and develop novel treatments and to complement the diagnostic algorithm.

2. For Ukraine: it is recommended to promote programs of routine screening of preinvasive disease with HPV and HSV detection and assessment immunity status; introduction of personalized outpatient gynecological care with high level of efficiency and patient safety; the introduction of ambulatory (‘office’) care as the patient-centered medical home (PCMH) model for health care delivery; to participate in project and in partnership with EU to follow up experimental and clinical trials and involve related institutions and centers to the study.

## Abbreviations

AR: Androgen receptor; CC: Cervical cancer; CVF: *Cervical vaginal fluid*; ER: Estrogen receptor; HPV: Human papillomavirus; MRI: Magnetic resonance imaging; MRS: Magnetic resonance spectroscopy; PA: Progesterone receptor antagonists; PET-CT: Positron emission tomography-computed tomography; PPPM: Predictive preventive, and personalized medicine; PR: Progesterone receptor; US: Ultrasound.

## Competing interests

The authors declare that they have no competing interests.

## Authors’ contributions

LML did the planning of the research, study of pro- and anti-inflammatory cytokines, data analysis, statistical data processing and prepared the article. OEN did the collection and analysis of clinical material and determination of cytokine production. EVN did the collection and analysis of clinical material and the types of papillomaviruses. OMD did the collection and analysis of clinical material of colposcopic examination of patients. GVK participated in the creation of test systems for the determination of the avidity of the IgG antibodies. LOG participated in the creation of test systems for the determination of IgG and IgM antibodies in the serum of patients. RVB did the ultrasound survey, performed and did the analysis of the study and the literature review in part of the biomarker panel development, formulated prospects and performed the final article drafting. VOS determined the presence of IgM and IgG antibodies in the serum of patients. NMN did the collection and analysis of clinical material of cytomorphological study of the cervical material. VVB did the collection and analysis of clinical material and diagnosis of genital herpes infection. MYS did the organization and analysis of the study, planning studies, analysis of data, and prepared the article. All authors read and approved the final manuscript.

## Authors’ information

LLM, Ph.D., D.Sci. Professor is a researcher in the Inteferon Department of Zabolotny Institute of Microbiology and Virology, National Academy of Sciences of Ukraine. OEN, M.D., is a medical doctor and fellow in the Odessa National Medical University. OMD, M.D., Ph.D., is a medical doctor in the Kyiv Perinatal Center. EVN, M.D., D.Sc., Professor, Department of Infectious Diseases Epidemiology Course Odessa National Medical University. EVN, Ph.D., is a medical doctor in the Kyiv Perinatal Center. GVK, M.S., fellow JSC SPC ‘DiaprofMed’. LOG, Ph.D., is a researcher of the Inteferon Department of Zabolotny Institute of Microbiology and Virology, National Academy of Sciences of Ukraine. RVB, M.D., Ph.D., is a researcher of the Inteferon Department of Zabolotny Institute of Microbiology and Virology, National Academy of Sciences of Ukraine and a medical doctor in the Clinical Hospital ‘Pheophania’ of the State Affairs Department, National Representative of the European Association for Predictive, Preventive and Personalised Medicine (EPMA) in Ukraine. VOS is a researcher of the Inteferon Department of Zabolotny Institute of Microbiology and Virology, National Academy of Sciences of Ukraine. NMN, M.D., is a medical doctor and fellow in the Odessa National Medical University. VVB, M.D., Ph.D., director of the Kyiv Perinatal Center. MYS, Ph.D., D.Sci., Professor is a corresponding member of the National Academy of Sciences of Ukraine and the director of the Inteferon Department of Zabolotny Institute of Microbiology and Virology, NAS of Ukraine, Kyiv, Ukraine.
